# Investigating Stress Limitations in Dynamic Response of Coral Limestone Concrete: Integrated FDM-DEM Simulations and Experimental Validation

**DOI:** 10.3390/ma18102268

**Published:** 2025-05-13

**Authors:** Yuzhu Zhang, Haoran Hu, Yi Luo, Yi Gong, Jinrui Zhang

**Affiliations:** 1 Changjiang Survey, Planning, Design and Research Co., Ltd., Wuhan 430010, China; 2 School of Civil Engineering and Architecture, Wuhan University of Technology, Wuhan 430070, China; 3 Sanya Science and Education Innovation Park, Wuhan University of Technology, Sanya 572024, China; 4 Housing and Transportation Bureau of Xinpu New District, Zunyi 563000, China

**Keywords:** FDM-DEM, concrete, SHPB, dynamic mechanics

## Abstract

This study established a dynamic impact simulation system for a coral limestone cement composite subjected to bidirectional stress confinement conditions by using a coupled method of continuous medium FDM (*a coupled continuum-discontinuum approach integrating finite difference continuum modeling (FDM) and the discrete element method (DEM) granular analysis*), and verified its accuracy through indoor experiments. The study first conducted dynamic mechanical performance tests on reef limestone concrete using an SHPB experimental device, exploring the effects of the *strain-rate governed high-rate response*, energy evolution, and failure modes. Subsequently, an FDM-DEM coupled model was used to simulate the impact-induced behavior of concrete at multiaxial stress conditions and constraint conditions, analyzing the strain-rate dependent performance of concrete exposed to biaxial monotonic loading. Test outcomes indicate that the increase in strain rate significantly enhanced the dynamic peak stress, and the collapse behavior shifted from type I to type II. As static loading in the σ_2_ direction increased, the dynamic peak stress in the σ_1_ direction decreased, while the dynamic peak stress in the σ_2_ direction increased, indicating that the constraint stress in the σ_2_ direction had an inhibitory effect on the sample’s failure. Through the time-history monitoring and analysis of cracks, it was found that the internal crack growth rate accelerated as the stress increased, while the crack growth tended to stabilize when the stress decreased. Additionally, this study explored the effect of stress constraints on the fragmentation patterns, revealing changes in the failure modes and crack distributions of the sample under different stress states, providing a theoretical basis and technical support for island and reef construction and engineering protection.

## 1. Introduction

In the construction of offshore islands and reefs, the construction of infrastructure mainly relies on the use of concrete [1,2]. However, concrete aggregates rely on the long-distance transportation of land-based building materials, which faces high costs and logistics bottlenecks [3]. Coral aggregates, which are locally sourced, provide a better solution for the localization of island and reef engineering materials due to their light weight, diverse surface geometric characteristics, and strong water absorption properties.

The reef limestone concrete engineering structures in island and reef projects not only have to withstand natural dynamic loads such as earthquakes, tsunamis, and typhoons, but also have to deal with the threat of strong dynamic loads such as impacts, heavy vehicle collisions, and penetration loads [4,5,6,7]. Taking tunnel lining as an example, its curved top plate needs to resist the biaxial static stress field formed by the uneven extrusion of the strata and the periodic dynamic wave disturbance caused by the passage of heavy vehicles during its service [8,9,10]. This dynamic and static superposition mechanical environment makes the evolution of microcracks inside the concrete show significant path dependence. Compared with the uniaxial loading mode, the biaxial constraint condition changes the propagation direction of the main crack, forcing the newly generated cracks to produce a bifurcation effect at the interface between the reef limestone aggregate and the cement matrix. The dynamic-static load coupling effect borne by the concrete structure presents an unprecedented complexity. The study of its dynamic mechanical behavior under biaxial constraints is of special essential relevance to the risk mitigation design of key structures such as bridge seismic columns and tunnel linings. Over the past decade, significant academic focus has been directed toward the potential applications of reef limestone concrete and explored its application value in terms of high performance and high strength. In terms of static mechanical tests, Sun Baolai et al. [11] effectively improved the uniaxial compressive strength of concrete by adding silica fume and proposed the optimal silica fume dosage, while Yuan Yinfeng [12] conducted a comprehensive study on the preparation method of reef limestone aggregate seawater concrete and provided quantitative indicators for the optimized mix ratio and mechanical properties. Mi Renjie et al. [13] demonstrated proportional correlations among the axial compressive strength, splitting tensile strength, flexural strength, and cube compressive strength in reef limestone seawater concrete, with corresponding linear formulas established. Chengjun Yue [14] studied the stress–strain curve of reef limestone concrete, calculated the static elastic modulus and Poisson’s ratio based on the relevant mathematical relationship, and found that compared with traditional crushed stone river sand concrete, reef-based cementitious materials were accentuated, while concrete exhibited more obvious brittleness. In terms of dynamic mechanical tests, researchers have employed split-Hopkinson bar (SHPB) experiments to evaluate the dynamic behavior of coral limestone concrete under high-rate loading, and all believe that coral limestone concrete is more sensitive to the rate effects in contrast to traditional binder systems [15,16,17]. Zhang Min et al. [4] and Wu Jiawen et al. [18] assessed the strain-rate dependent behavior of biogenic aggregate concrete by employing Hopkinson compression bars (75 mm and 100 mm). Their results showed that the dynamic strength growth coefficient of reef limestone concrete was proportional to the strain rate to the power of 0.5, and its strength growth coefficient was higher than that of similar ordinary silicate cement. Qin et al. [19] took coral reef sand concrete as the research object and conducted research on the impact-induced behavior, energy propagation metrics, and the failure morphology and failure mechanism under impact loading. At present, the research on reef limestone concrete has mainly focused on static and one-dimensional dynamic mechanical tests, such as uniaxial compression and simple one-dimensional impact experiments, which cannot simulate the complex biaxial static pressure and dynamic impact conditions in marine engineering. Majid Movahedi Rad [20] summarized the theoretical and application progress of elastic-plastic stability analysis under finite plastic deformation and displacement constraints, covering the extension of the stability theorem, numerical algorithms, and their application in the safety assessment of cyclic load structures. Vahid Shafaie [21,22] proposed a multi-objective genetic algorithm based on the discrete element method for optimizing the parameters of color self-compacting concrete, balancing the rheological properties and color uniformity, improving the design efficiency, and providing numerical support for the industrial production of high-performance decorative concrete. Through oblique shear tests combined with fuzzy logic, the shear bond strength of the SCC and FRSCC in repair engineering was systematically evaluated. The results indicated that fibers significantly improved the interfacial performance, while the fuzzy logic model achieved the dynamic prediction of strength, aiding in the assessment of structural durability.

With the proliferation of high-performance computing and predictive modeling tools, scholars at home and abroad have developed different mesoscopic simulation methods to conduct numerical studies on the mechanical properties. Feng WH et al. [23] constructed LS-DYNA-driven SHPB models to explore the strain-rate dependency in steel-reinforced concrete under dynamic compression; Qiu H et al. [24] applied AUTODYN simulations for interfacial fracture and stress wave propagation in rock-mortar bimaterial systems; and Shi DD et al. [25] utilized ABAQUS-based FEM to evaluate the impact resistance of sustainable concrete composites with recycled aggregates.

Therefore, it is more reasonable to construct numerical models by using the continuous medium model and discrete medium simulation to simulate the rod system and rock samples, respectively. In addition, when only the discrete element method is used to simulate the SHPB experiment, the force transmission of the entire system is completely borne by the “contact”. A large number of contact calculations make the computer workload heavy, and the gaps between particles also cause certain deviations in the calculation process. The FDM-DEM coupled model construction method can effectively optimize these deficiencies and significantly enhance the calculation efficiency. However, this coupling method is rarely used in impact dynamics experiments simulating concrete.

In light of this, we established a two-dimensional numerical model of the separated Hopkinson compression bar based on the continuous (FDM finite difference method)-(DEM discrete element method) coupling method. Through comparative analysis with indoor experimental results, the rationality and reliability of the model were verified. The deformation rate sensitivity in high-rate strength characteristics growth, energy evolution, and the failure mode of reef limestone concrete was further studied in order to provide a theoretical basis and technical support for island and reef construction and engineering protection.

## 2. Dynamic Impact Test Study of Reef Limestone Concrete

### 2.1. Sample Preparation

**Figure 1:**
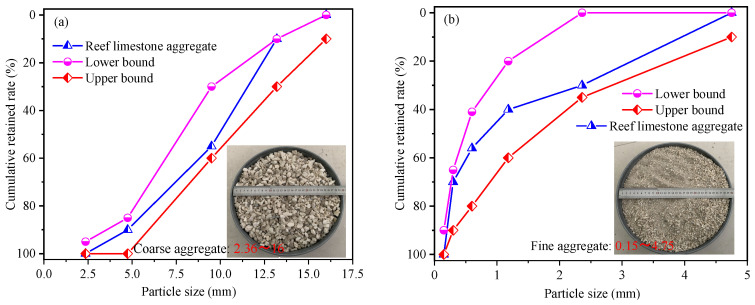
Reef tuff aggregate grading curve. (**a**) Particle size distribution map of coarse aggregate in reef limestone, (**b**) Particle size distribution map of fine aggregate in reef limestonewo.

The experimental aggregates were derived from South China Sea coral reef formations. The large pieces of reef limestone were crushed using a PE111X150-1 crusher (Hai’an Eternal Vibration Machinery Co., Ltd. Qutangdong Industrial Park, Nantong City, Jiangsu Province, China), and the particle size at the crusher’s outlet was 5–25 mm. Due to the low strength of reef limestone, more powdery impurities will be produced during the crushing process. Untreated coral-derived aggregates can destabilize concrete matrices. Following comminution, the crushed materials were graded into coarse (4.75–16 mm) and fine (0.15–4.75 mm) fractions. Figure 1 illustrates the continuous gradation profile of these marine-origin aggregates.

C30-grade concrete was formulated using reef-derived aggregates (physical parameters in Table 1), Portland cement, water, and supplementary cementitious materials (fly ash/slag) blended per the proportioning scheme in Table 2.

Reef limestone aggregates were immersed in deionized water for 12 h pre-mixing to purge ionic impurities. A polycarboxylate high-range water reducer was introduced during blending to minimize shrinkage-induced cracking and optimize durability. The mixed compound was poured into molds, mechanically compacted via vibration, and stripped post 24-h hardening. Specimens underwent 28-day curing under controlled conditions (20 ± 2 °C, RH ≥ 95%).

### 2.2. SHPB Device and Principle

**Figure 2:**
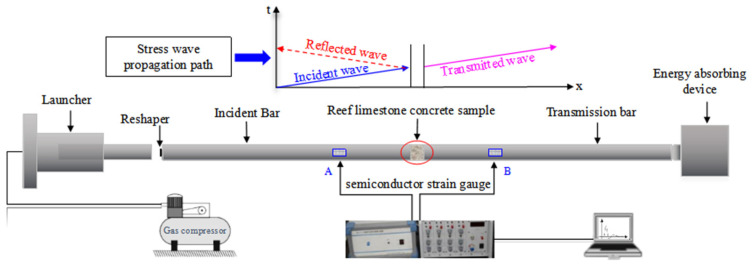
SHPB impact test system.

The SHPB experiments on reef limestone concrete were based on the ASTM D7012 [26] rock dynamic fracture toughness test. Dynamic impact characterization utilized a split-Hopkinson pressure bar (SHPB) system, as depicted in Figure 2. The device mainly consists of three parts: the impact projectile, incident bar, and transmission bar (Figure 2). The system incorporates three core components: a striker bar, incident bar, and output bar, with lengths of 0.4 m, 2.8 m, and 2.8 m, respectively, and a bar diameter of 60 mm. During the test, the power device first drives the impact head to impact the incident rod, thereby generating an incident stress wave that propagates along the axial direction. When the incident wave propagates upon stress wave propagation from the incident bar’s end to the specimen contact surface, acoustic impedance disparity at the bar–specimen interface generates reflected/transmitted waves. Part of the incident stress wave is converted into a reflected wave, and the other part is transmitted through the reef limestone concrete pattern to the transmission rod in the form of a transmitted wave. In this process, the incident wave, reflected wave, and transmitted wave are measured by the resistance strain gauges attached to the incident rod and the transmission rod. The metal bar demonstrated a Young’s modulus of 205.6 GPa, mass density of 7.767 × 10^3^ kg/m^3^, and longitudinal wave speed of 5145 m/s. The SHPB dynamic stress calculations adhere to the 1D elastic wave propagation theory and specimen strain homogeneity principle. Following 1D stress wave mechanics, impact-induced dynamic parameters (*σ*, *ε_i_*, $\dot{\varepsilon }$*_r_*, *ε_t_*) for marine aggregate concrete were computed as follows:

(1) $$\begin{array}{l} \sigma_{t}=\frac{\left(\varepsilon_{i}\left(t\right)+\varepsilon_{r}\left(t\right)+\varepsilon_{t}\left(t\right)\right)E_{0}A}{2A_{s}}\\ \dot{\varepsilon }\left(t\right)=\frac{\left(\varepsilon_{i}\left(t\right)-\varepsilon_{r}\left(t\right)-\varepsilon_{t}\left(t\right)\right)C_{0}}{L_{s}}\\ \varepsilon \left(t\right)=\int_{0}^{t}\dot{\varepsilon }(t)dt \end{array}$$

where *E*_0_, *C*_0_ are the elastic modulus and elastic wave velocity of the compression rod, respectively; A is the cross-sectional area of the compression rod; *ε_i_*, *ε_r_*, *ε_t_* are the incident strain, reflected strain, and transmitted strain, respectively; *A_S_*, *L_S_* are the cross-sectional area and length of the specimen, respectively; and *t* is the duration of the stress wave.

### 2.3. Test Results Analysis

**Figure 3:**
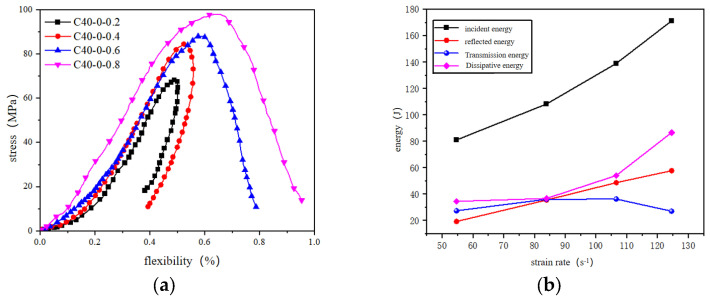
Typical dynamic stress–strain curve. (**a**) Dynamic stress-strain curve of reef limestone concrete. (**b**) Energy variation curve of reef limestone concrete.

Under applied gas pressures of 0.2–0.8 MPa, Figure 3 presents the stress–strain responses of the reef limestone concrete under varied strain rates. The curves exhibited four distinct phases: the compaction phase, elastic regime, plastic hardening stage, and post-peak softening. High strain-rate loading accelerates compaction completion, rendering this phase indistinct in stress–strain curves. Specimens demonstrated strain-rate strengthening effects, with dynamic strength exhibiting a positive correlation to the loading velocity. Elevated strain rates induced progressive steepening of the pre-peak stress-strain profiles, demonstrating amplified slope gradients and enhanced Young’s modulus in both material states. Table 3 shows the failure modes of reef limestone concrete under different strain rates.

## 3. Multiscale Simulation of Dynamic Loading Effects on Reef Aggregates Using FDM-DEM Coupling

### 3.1. Numerical Model Establishment

A hybrid FDM-DEM numerical framework was implemented to simulate the SHPB behavior of the reef limestone concrete. The FLAC2D-PFC2D coupling mechanism operates through boundary-controlled wall-mediated continuum-discrete interaction. During each PFC2D computational iteration, particle interactions are governed by force-displacement constitutive laws at discrete contacts, while particle kinematics adhere to the principles of Newtonian dynamics. Granular assemblies and confinement walls exhibit dynamic positional updating per computational cycle. The simulation of impact loads in FLAC2D is centered around pressure driving, and dynamic loading is achieved by defining stress wave time history functions, rather than directly relying on velocity or experimental strain data.

**Figure 4:**
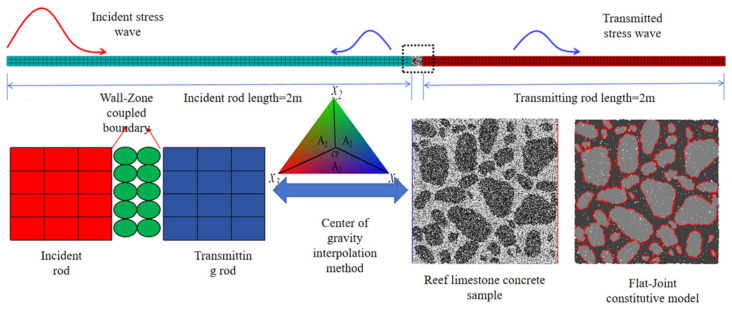
Numerical model of SHPB based on the FDM-DEM coupling method. The dashed box represents the comparison between the microscopic image of reef limestone concrete and the model simulation parameters selected for numerical simulation.

Based on the above theory, the SHPB compression rod system and reef limestone concrete were replicated in a 1:1 ratio for indoor tests. The compression rod system is a continuous medium constructed by the finite difference of FLAC2D and adopts a linear elastic model constitutive method. By assigning mechanical parameters such as the elastic modulus and Poisson’s ratio, the properties of the loading system are equivalent to the properties of steel, and there is no loss in the generation and propagation of elastic stress waves. The reef limestone concrete style was simulated by the discrete element medium constructed by PFC. The contact surface composed of triangular walls was established by the “wall-zone” command as the coupling interface between FLAC2D and PFC2D. The stress wave propagated in the rod was calculated by the centroid interpolation method through the contact surface nodes, and acts on the particles at the contact surface to complete the whole process of stress wave incidence, reflection and transmission, as shown in the Figure 4.Therefore, the continuous-discrete coupling modeling method is more likely to meet the “stress uniformity assumption” of stress wave propagation in SHPB tests. In addition, the PFC particle constitutive model selects the flat-joint model (as shown in Figure 4). This methodology effectively captures microcrack nucleation during loading and macrocrack coalescence, with interparticle tensile strength thresholds calibrated at grain boundaries. If the introduced stress is below this threshold, the particles maintain close contact; once the stress exceeds the strength limit, the bond will break, and the bond break between the sample particles is considered to have generated microcracks. The area where the microcracks severely expand is often accompanied by particle shedding and the formation of fragments.

**Figure 5:**
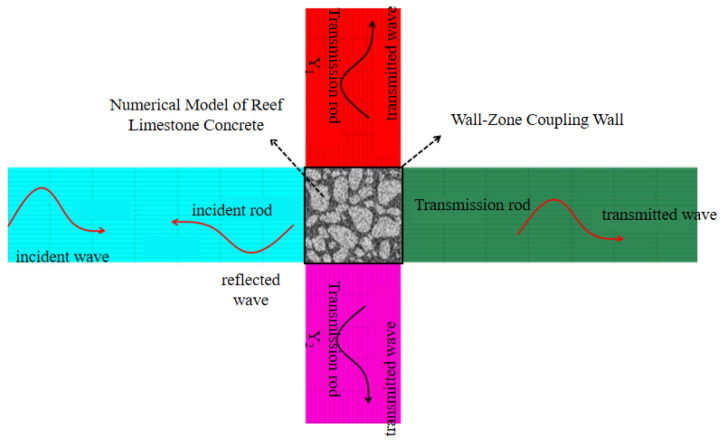
Numerical model of the reef limestone concrete under biaxial stress constraint conditions.

A numerical model of the reef limestone concrete under biaxial stress constraint conditions was established, as shown in Figure 5:

The model consisted of zone units to form transmission rods Y1 and Y2. The contact ends of the rods and the specimens were connected by coupling walls, which ensured the mechanical connection with the particle model. During the modeling process, the width of the rods in the X direction needs to be controlled. A distance of 5 mm was also reserved in the model to prevent stress concentration during stress wave loading, which may cause model errors.

Stress wave transmission dynamics within the numerical framework initiate as the incident pulse propagates along the input bar toward the specimen’s proximal interface. Due to the existence of wave impedance, part of the stress wave is emitted to form a reflected stress wave, and part of it passes through the sample and then enters the transmission rod through the coupling wall to form a transmitted wave. The transmission stress wave is generated in the Y-axis direction due to the Poisson effect and propagates from the upper and lower surfaces of the sample to the two ends of the Y-axis.

During the calculation process, the stress wave is input at the end of the incident rod, and the discrete fracture grid (DFN) image reflects the crack evolution characteristics of the sample. When the bonding between the individual particle entity and the surrounding is broken, segmentation cracks are generated and fragments are produced, which characterize the fragments (debris) produced by the sample destruction, thus better reflecting the damage and failure characteristics of reef limestone concrete.

**Figure 6:**
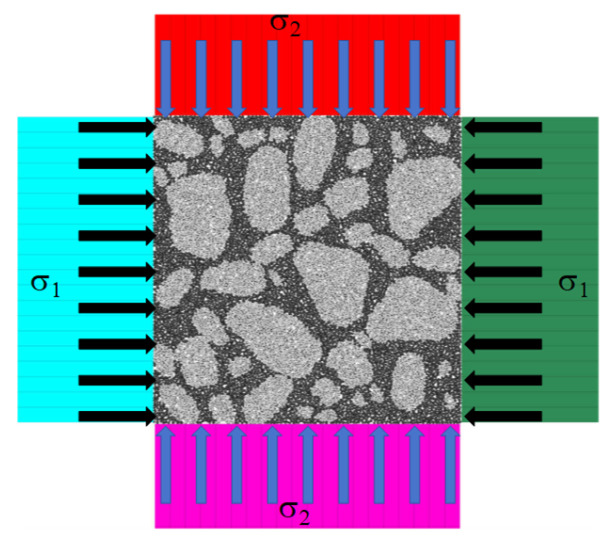
Confining pressure loading of the numerical model.

**Figure 7:**
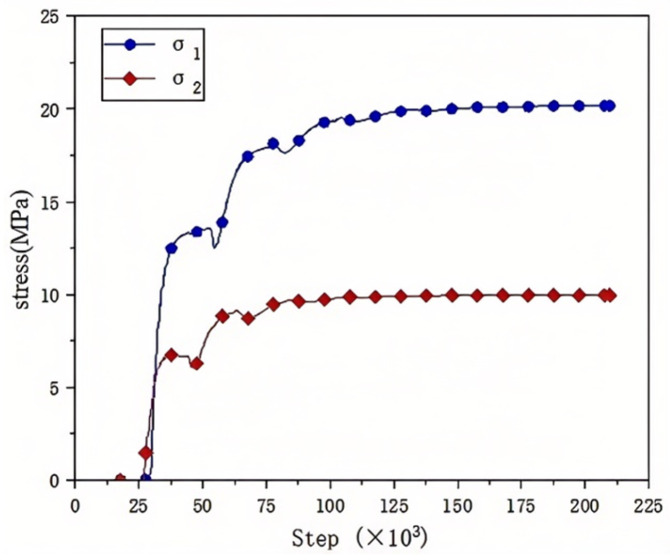
Confining pressure loading curve.

Figure 6 shows the confining pressure loading of the numerical model of reef limestone concrete. The command used for confining pressure loading was zone face apply, and loading was performed at the ends of the four rods away from the specimen. The stress was transmitted to the inside of the specimen through the rod. With the specimen width as the diameter, a measuring circle was set at the center of the specimen to monitor the internal stress state of the specimen at all times. As shown in Figure 7, σ_1_ and σ_2_ were the confining pressure–time step time history curves in the X and Y directions, respectively. It can be seen from the figure that due to the short length of the Y-axis transmission rod, the confining pressure stress reached the specimen faster, and the starting point was forward. In addition, due to the existence of wave impedance, the confining pressure stress will be reflected after reaching the specimen, resulting in stress fluctuations inside the specimen. When the fluctuation ends, the final internal stress of the specimen tends to be the target confining pressure value. When the internal stress of the model is balanced, the dynamic load input is performed.

### 3.2. Microscopic Parameter Calibration and Correction

The calibration of microscopic parameters in the numerical simulation of granular flow usually adopts the “trial and error method”, which has achieved good results [13,14]. Limited by the test conditions, the static constraint in the σ_2_ direction in the established biaxial reef limestone concrete numerical model was set to 0, and numerical simulations of the reef limestone concrete under two strain rate loading conditions were carried out and compared with the results of the conventional indoor SHPB impact test above-mentioned. It can be seen from Table 4 that the yield strength and crushing state of the impact test and the corresponding particle flow numerical simulation test were similar, and the two were quite consistent, which verifies the reliability of the created particle flow model.

Laboratory-derived experimental data guided the iterative adjustment of the FDM-DEM coupling parameters, culminating in the finalized material and bar assembly properties tabulated in Table 5 and Table 6.

### 3.3. Stress Balance Verification Analysis During Dynamic Impact Process

**Figure 8:**
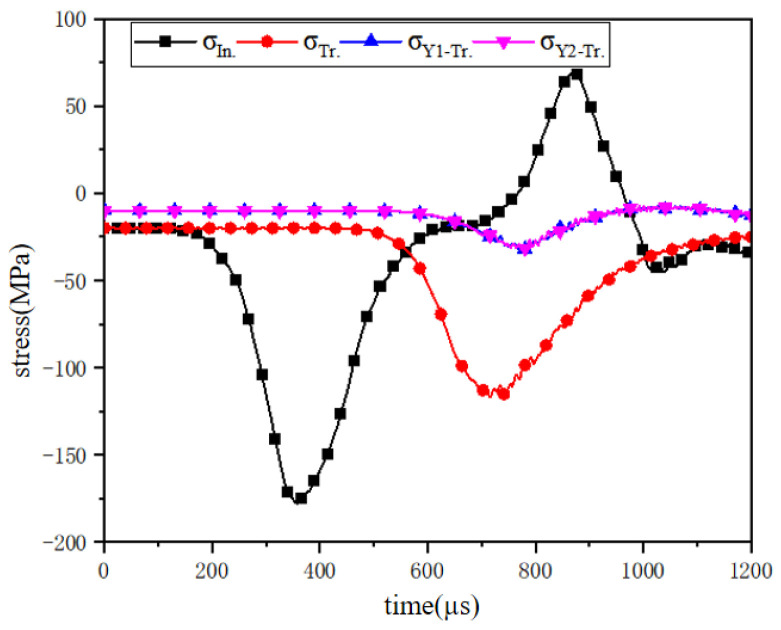
Stress–time history curve.

Figure 8 shows the waveform of the numerical model of the reef limestone concrete under a pre-stress of (20,10) MPa. Since the measuring point on the rod was at a certain distance from the dynamic load, the incident stress wave was always 20 MPa at 200 μs. When the time exceeded 200 μs, the stress value began to increase. When the stress value reached about 175 MPa, the stress value began to decrease. Finally, at about 600 μs, the incident wave completed the propagation process. Then, the transmitted wave began to increase. Due to the limited strength of the sample model, the transmitted wave finally began to decrease at about 125 MPa, and the surface stress wave transmission process ended. The peak value of the transmitted stress wave in the Y-axis direction was approximately 25 MPa, which was mainly due to the Poisson effect of the particle model. As the stress wave propagated, the stress wave in the Y-axis direction eventually approached the confining stress value. In general, the reflection and transmission process of the stress wave could be clearly seen from the figure, indicating that the model had high reliability in the stress propagation process.

**Figure 9:**
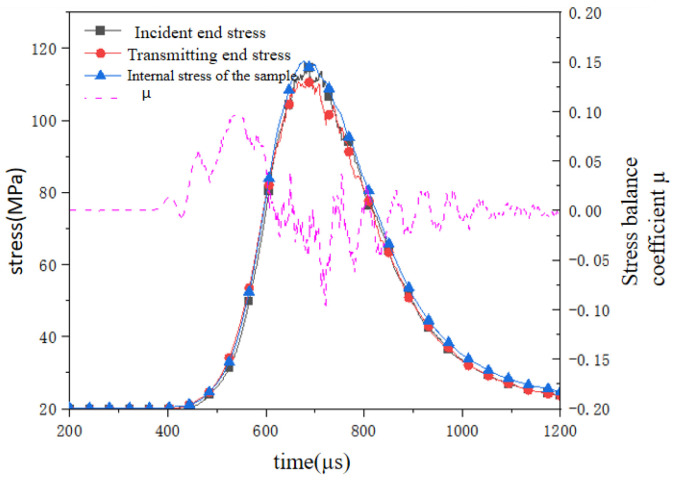
Balance detection.

Similarly, evaluating the dynamic stress balance of the reef limestone concrete under biaxial stress constraints is a necessary condition for measuring the accuracy of the numerical simulation. Calculated by Formula (2), the stress state at both ends of the specimen and inside is shown in Figure 9. The incident end stress and the transmission end stress of the particle model basically coincided before 700 μs. There was a more drastic stress fluctuation at 700–800 μs, which may have been the result of the combined effect of static stress and the dynamic stress wave of confining pressure. At this stage, the stress balance coefficient (μ) showed the same fluctuation trend, but the overall value fluctuated within ±5%. When the time reached 1000 μs, the internal stress of the sample reached equilibrium again, and finally the three simultaneously approached 20 MPa. The stress balance coefficient fluctuated around the zero value and quickly approached zero. The experimental validation confirmed that the particle-based specimen maintained adequate stress equilibrium at both termini throughout dynamic loading, thereby fulfilling the prerequisite of stress homogenization under transient conditions.

(2) $$\tau_{f}=c_{f}-\bar{\sigma }\tan\varphi_{f}$$

(3) $$\mu =\frac{2\left(\sigma_{In}-\sigma_{Tr}\right)}{\left(\sigma_{In}+\sigma_{Tr}\right)}$$

In the formula, $c_{f}$, $\varphi_{f}$, $\bar{\sigma }$, and $\mu_{f}$ represent the cohesive force, friction angle, and normal compressive stress acting on the unit cell ball, respectively.

Based on this, the FDM-DEM coupled biaxial constraint model had good reliability, and the stress constraint conditions of the design calculation conditions were (5,10), (10,10), (15,10), (20,5), (20,10), (20,15), (20,20), and the three kinds of impact loads with equal gradient increases of 0.3 MPa, 0.45 MPa, 0.6 MPa, 0.75 MPa, 0.9 MPa. This numerical framework enabled the systematic investigation of the coral limestone concrete’s dynamic behavior through the quantitative assessment of the σ_1_-axis stress–strain response under impact loading, the dynamic response in the σ_2_ direction, the variation law of peak strain, and the typical failure characteristics of the specimens.

### 3.4. Results and Discussion

#### 3.4.1. Effect of Biaxial Stress Constraint on Dynamic Mechanical Properties

**Figure 10:**
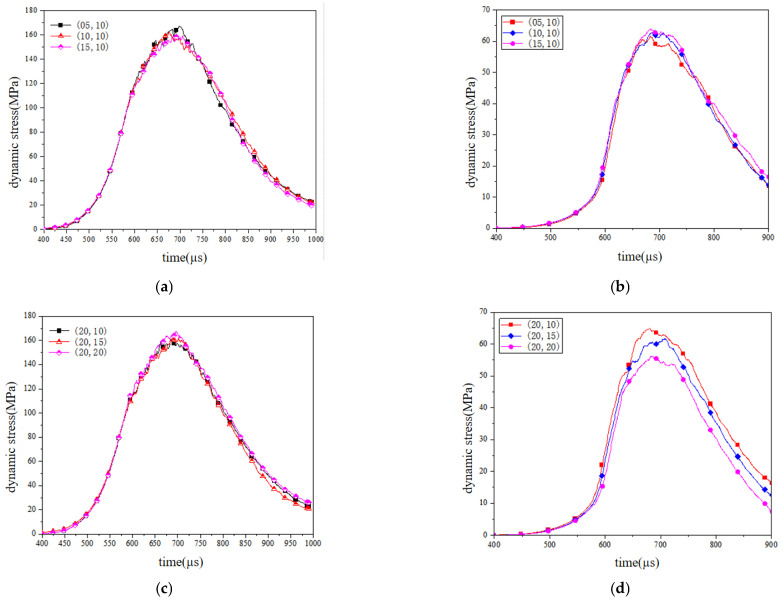
Dynamic stress–strain curves under different stress constraints. (**a**) X-axis static load-X-axis dynamic stress–time history curve. (**b**) X-axis static load-Y-axis dynamic stress–time history curve. (**c**) Y-axis static load-X-axis dynamic stress–time history curve. (**d**) Y-axis static load-Y-axis dynamic stress–time history curve.

Figure 10 shows the dynamic stress–time history curves obtained by setting three static load conditions of equal gradient in the X and Y directions of the numerical model of reef limestone concrete and calculating them under the same impact load.

Analysis of the figure indicated a comparable impact loading conditions yield (0.6 MPa), and a comparative analysis of the working conditions (05,10), (10,10) and (20,10) in Figure 10a showed that when the initial static load in the σ_1_ direction increased from 5 MPa and 10 MPa to 15 MPa, the dynamic peak stress in the σ_1_ direction decreased from 166.90 MPa and 162.42 MPa to 159.24 MPa, respectively; the corresponding dynamic peak stress in the σ_2_ direction increased from 61.62 MPa and 62.85 MPa to 64.95 MPa, respectively. Experimental observations revealed that under equivalent dynamic loading conditions, elevating the static confining pressure along the σ_1_-axis induced a marginal attenuation of the dynamic peak stress in the same orientation, demonstrating a strain-rate-sensitive stress suppression phenomenon. Conversely, augmenting static loads in the σ_2_-axis elicited a moderate amplification of dynamic stress peaks along the orthogonal axis.

Through a comparative analysis of the strain-rate-dependent mechanical responses across loading configurations (20,10), (20,15) and (20,20), it can be seen that when the initial static load in the σ_2_ direction increased from 10 MPa to 20 MPa, the dynamic peak stress of the particle model in the impact direction increased from 160.87 MPa to 167.77 MPa, while the dynamic peak stress in the σ_2_ direction decreased from 64.98 MPa to 56.23 MPa.

Under identical impact loading protocols, an elevated σ_2_-axis static confinement pressure exerts pronounced directional modulation on dynamic mechanical responses. Specifically, σ_1_-orientation peak dynamic stress demonstrated monotonic escalation with increasing σ_2_ static loads, whereas σ_2_-direction dynamic stress maxima exhibited progressive attenuation under such multiaxial stress coupling configurations.

In summary, it can be concluded that the dynamic performance of the numerical model of reef limestone concrete under biaxial static load conditions is closely related to the size of its initial static load. Under the condition of the same static load in the σ_1_ direction, the increase in the static load in the σ_2_ direction significantly increased the dynamic peak stress in the σ_1_ direction and significantly reduced the dynamic peak stress in the σ_2_ direction.

**Figure 11:**
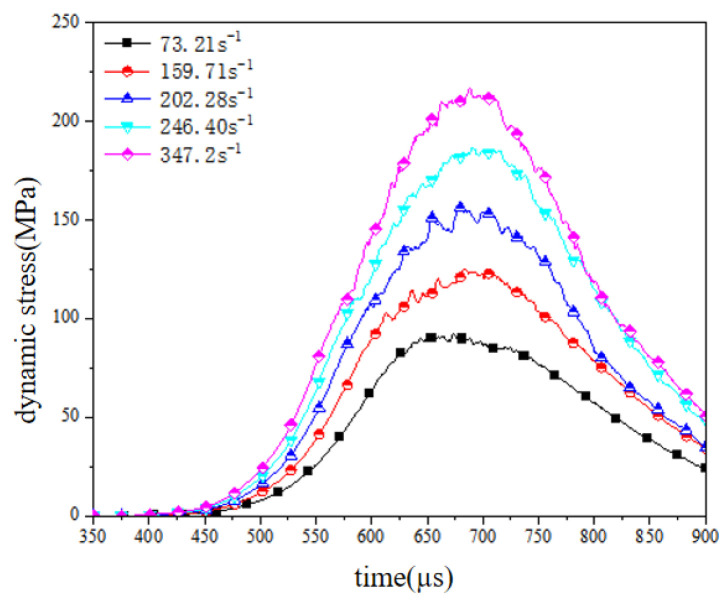
Dynamic stress–time history curve.

Furthermore, to quantitatively assess the strain-rate sensitivity in coral limestone concrete under biaxial confinement, numerical simulations were executed with fixed prestress boundary conditions (σ_1_ = 20 MPa, σ_2_ = 5 MPa) while applying impulsive pressures incrementally from 0.3 MPa to 0.9 MPa (Δ = 0.15 MPa intervals). Figure 11 shows the dynamic stress–time history curve of the numerical simulation calculation results. The magnitude of the impact load had a significant effect on the shape of the starting stage of the stress–time history curve in the X-axis direction. Under a low load, the stress wave energy was low and the propagation speed in the rod was slow. Therefore, the dynamic stress of the sample increased slowly in the early stage.

When the dynamic stress magnitude remained below the interfacial bond capacity threshold of the numerical specimen, the model retained structural integrity with only localized fracture nucleation. The stored strain energy underwent rapid dissipation during the post-critical regime, manifested as a gradual stress decay phase (unloading gradient < 15 MPa/ms) in the temporal stress profile. Due to the distributed microcrack networks generated by impact-induced damage evolution [25,27], irreversible residual strain persisted even after full stress relaxation.

**Figure 12:**
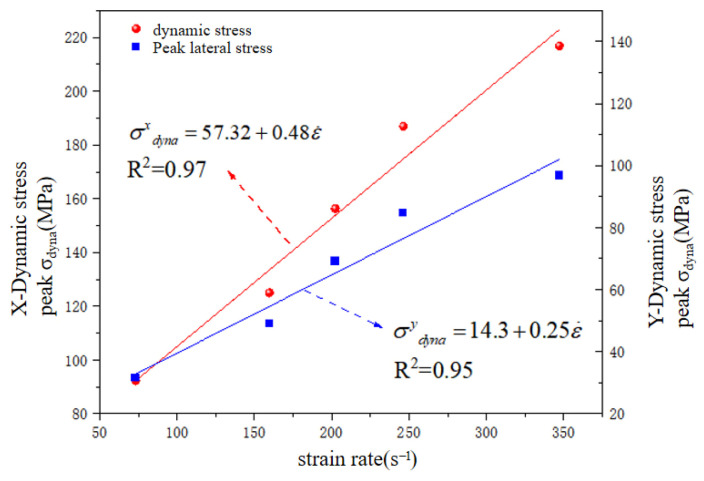
Strain rate–dynamic stress curve.

When the dynamic stress amplitude exceeds the interfacial strength threshold of the discrete element assembly, the system undergoes irreversible damage characterized by accelerated post-failure stress relaxation kinetics. Figure 12 quantitatively captures the strain-rate dependency of the coral limestone concrete’s biaxial dynamic response, revealing a linear proportionality between the strain-rate intensification (from 73.21 s^−1^ to 347.2 s^−1^) and dynamic strength amplification. Specifically, the X-axis compressive strength escalated from 92.29 MPa to 216.91 MPa (the growth rate reached 135.03%), while the Y-axis stress exhibited an analogous rate sensitivity with 88.7% intensification, both demonstrating strong correlation coefficients (R^2^ ≥ 0.94). This strain-rate hardening phenomenon, driven by microcrack coalescence dynamics during high-rate loading [28], manifested accelerated unloading phase transitions (α = 2.3–4.7 acceleration factors) and aligned with the conventional split-Hopkinson pressure bar (SHPB) experimental trends, thereby validating the numerical framework’s capacity to replicate rate-dependent fracture mechanics in heterogeneous geomaterials under multiaxial confinement.

#### 3.4.2. Dynamic Damage and Mesoscopic Fracture Evolution Under Biaxial Stress Constraints

**Figure 13:**
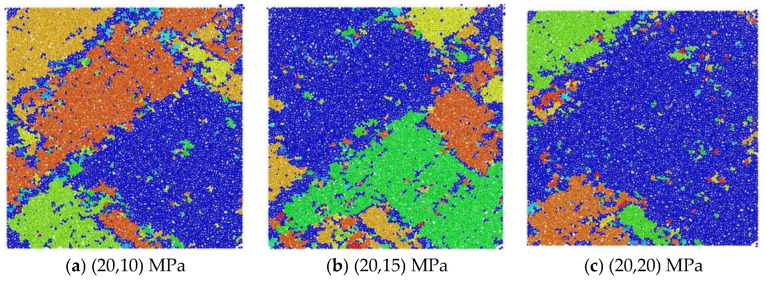
The broken (20,σ_2_) stress constraint model.

Figure 13 delineates the failure morphology of the coral limestone concrete in numerical simulations under multiaxial confinement scenarios. The model was in the (20,σ_2_) MPa state. Under the same impact load, as the value of σ_2_ increased in equal gradients, the crushing state of the sample changed significantly. When the stress state was (20,10) MPa, the main fracture area of the sample was the upper left corner of the model, and the fracture area was mainly composed of four types of fragments. The lower right corner of the sample had good integrity, but there was a small amount of debris. When the stress state increased to (20,15) MPa, the crushing condition at this time was the opposite to the previous one. The crushing area was mainly concentrated in the lower right corner, which was mainly composed of three main fragments. The overall crushing degree was reduced, but the crushing condition at the bottom of the model was more severe than before. As the stress in the Y direction continued to increase, when the stress state of the model reached (20,20) MPa, the computational results demonstrated a marked attenuation in the model fragmentation intensity, evidenced by an 83% reduction in the debris volume and fracture localization predominantly within the upper-left and lower-left quadrants. These primary fracture zones comprised two dominant failure blocks with fragment size distributions exhibiting a 56–72% reduction in equivalent diameter compared with the baseline fragmentation patterns. Therefore, through numerical simulation, we can see that the stress constraint value in the σ_2_ direction had an inhibitory effect on the degree of sample crushing. As the stress constraint value increased, the number of model cracks decreased by 10% and 30%, respectively. Therefore, the degree of internal crushing of the sample was significantly reduced.

**Figure 14:**
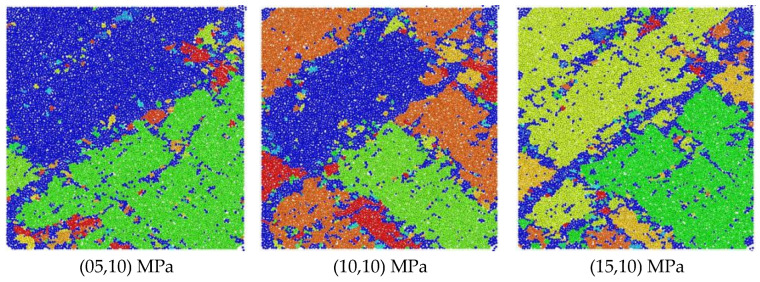
The broken (σ_1_,10) stress constraint model.

In addition, when the model specimen was in the state of (σ_1_,10) MPa, under the same impact load, the crushing of the test coupon was as shown in Figure 14. It can be preliminarily seen that the crushing of the model specimen had the opposite trend to that of the (20,σ_2_) MPa state. When the model specimen was in the stress constraint state of (5,10) MPa, under the impact load, the model test coupon was mainly broken into two larger fragments along the 45° direction, marked by blue and green, respectively. At this time, the integrity of the test coupon was better. As the stress in the impact direction increased by 5 MPa, the degree of crushing of the particle model after the dynamic load intensified, evolving from the previous two main fragments to six main fragments, and the main fragments were concentrated in the lower right and upper left corners of the model. When the constraint stress value reached the final 15 MPa, the degree of sample crushing was the most serious, with more debris, a smaller fragment size, and overall crushing failure. At this time, the specimen had a through crack on the oblique section. According to the analysis of elastic-plastic mechanics, this is because the yield strength of the oblique section is low under the bidirectional constraint condition. When the dynamic load exceeded the yield strength of the oblique section, a crack appeared on the oblique section. As the crack evolved, it eventually penetrated the entire model specimen. Therefore, in the (σ_1_,10) stress constraint state, the constraint stress in the impact direction promoted the crushing of the test coupon.

**Figure 15:**
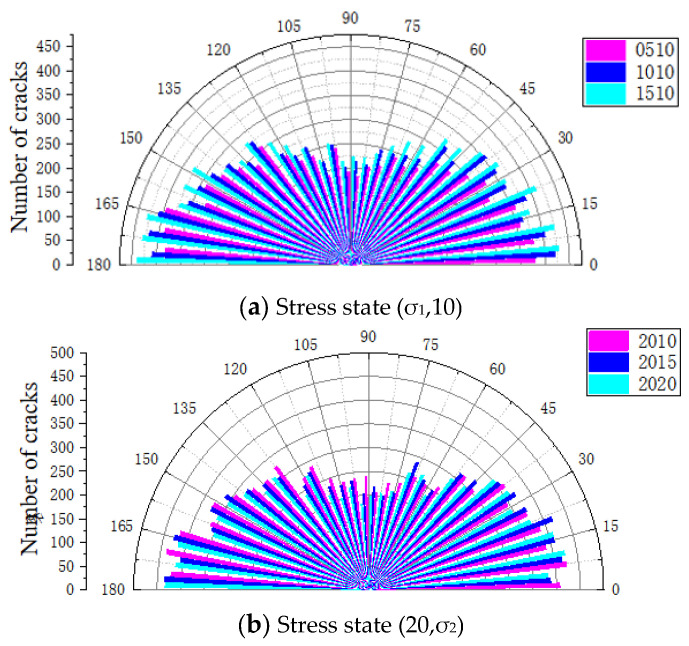
The (σ_1_,10) crack angle-number of models under different stress constraints.

Through the FISH language built into the PFC 6.00.30 software, a crack angle and quantity identification program was written, the angle and quantity of the cracks of the model specimen were counted and recorded using tables, etc., the data were output, and a crack distribution diagram after dynamic impact after the stress constraint value changes in two different directions was drawn as shown in Figure 15.

As shown in the figure above, when the particle model stress was in the (σ_1_,10) state, the comprehensive analysis indicates that the angle and number distribution of cracks were M-shaped, that is, cracks mainly appeared at 0~75° and 120~180°. The number of cracks in the 90° direction accounted for the lowest proportion. When σ_1_ = 5 MPa, the number of cracks in the direction around 165° was the largest, reaching about 390. The proportion of cracks between 105 and ~75° was the lowest, with an average number of less than 250. When σ_1_ = 10 MPa and σ_1_ = 20 MPa, cracks mostly appeared at 165~180°, and the number was roughly between 450 and ~425. Similarly, the lowest number also appeared in the 90° direction.

It is worth noting that with the increase in σ_1_, the crack density in any angle direction showed an upward trend. Under the three stress conditions, the cracks were concentrated in the range of 165~180°, indicating that the macro trend was mainly parallel to the impact direction. In addition, the number of cracks perpendicular to the impact direction was the least, which was due to the Y-axis stress constraint condition, which inhibited the displacement of the model specimen in the Y direction.

When the particle model stress was in the (20,σ_2_) state, the distribution of cracks with angles was generally consistent with the former, and both were M-shaped. When σ_2_ = 10 MPa, the model specimen had the most cracks in the 150~180° direction, with a number of about 450. In addition, between 0 and ~37.5°, the crack density was roughly the same, with no significant changes. Between 45 and ~105°, the crack density decreased significantly, and the number of cracks decreased to about 200. The same trend occurred at σ_2_ = 15 MPa and σ_2_ = 20 MPa. When σ_2_ = 15 MPa, the angle with the largest number of cracks was about 180° with a number of 436, and the least number was at 84° with a number of 201. When σ_2_ = 20 MPa, the cracks of the model were concentrated in the 12° and 180° directions with 425 and 434, respectively. In addition, with the increase in σ_2_, the crack density in each direction showed nonlinear changes. For example, in the 127° direction, the number was the largest when σ_2_ = 10 MPa, and the number was the least when σ_2_ = 15 MPa. At angles of 72° and 17°, the number of σ_2_ = 15 MPa was significantly higher than the other two. When σ_2_ = 20 MPa, the number of cracks did not increase or decrease significantly compared with the other two, and was mostly the same as the other two. This is consistent with the macroscopic level that when σ_2_ = 20 MPa, the overall crushing of the model specimen was reduced, and the specimen integrity was better.

**Figure 16:**
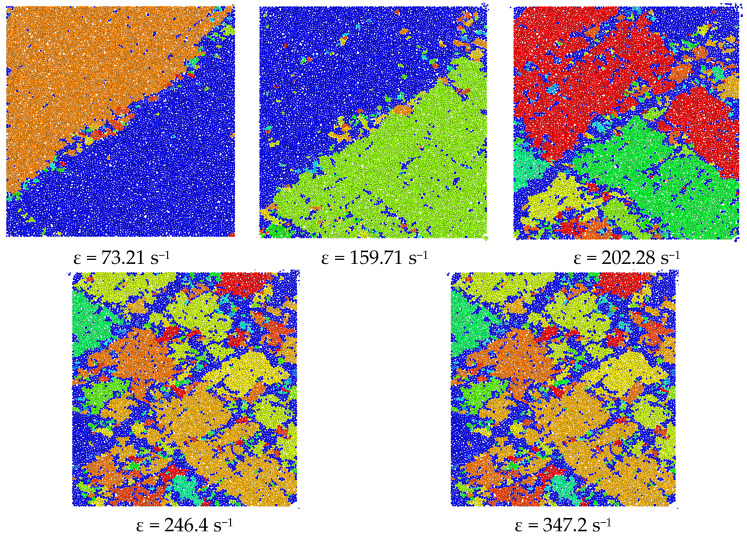
Model fragmentation diagram at different strain rates.

Figure 16 delineates the strain-rate-dependent fragmentation patterns of the numerical specimen, demonstrating progressive comminution intensification under elevated strain-rate loading. When the strain rate as 73.21 s^−1^, the integrity of the sample was good. Under the action of impact load, the sample underwent shear failure of the oblique section, and the interface was roughly about 40°. Due to the prismatic element’s presence within a multiaxial stress state, the lateral displacement of the sample was suppressed, resulting in the fragments still tightly biting together after the sample was broken. When the strain rate increased to 159.71 s^−1^, the sample was generally sheared, the crack surface was 45°, the total number of fragments remained unchanged, and it was composed of two main fragments. When the strain rate climbed to 202.28 s^−1^, the degree of fragmentation of the model sample intensified, the fragments were rectangular, and the number increased, consisting of five larger fragments. As the strain rate continued to rise, when the strain rate reached 246.4 s^−1^, the model sample was powdered after experiencing the impact load, and the sample crushing process was strong. At this time, the crushing mode of the fragments could no longer be seen. When the strain rate continued to climb to 347.2 s^−1^, the degree of sample crushing was slightly reduced but was generally powdery, the crack opening of the oblique section was increased, and the gaps were all debris particles. In general, the degree of crushing of the model sample tended to gradually rise with the increase in strain rate, but when the strain rate was 347.2 s^−1^, the degree of sample crushing was slightly reduced. The reason for this phenomenon may be that numerical simulation calculations have certain limitations: after the model specimen composed of ball units was loaded at an extremely high strain rate and broken into individual particles, the volumetric rigidity of discrete elements induced sustained strain-rate intensification, concurrently mitigating the progression of specimen comminution.

**Figure 17:**
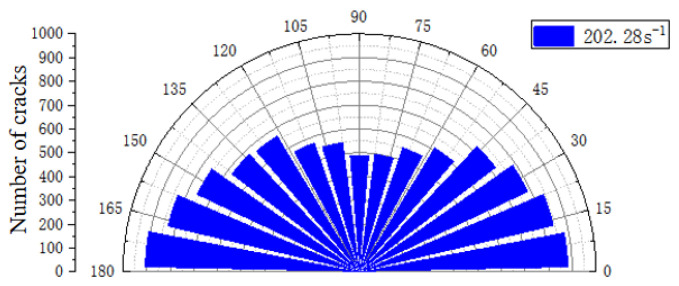
The ε = 246.4 s^−1^ crack distribution map.

Similarly, internal fractures in the reef limestone concrete discrete element model under varying strain rates were quantified through FISH language scripting. The rose drawing in this part took 12° as an interval length, with a total of fifteen intervals. Figure 17 is the result of the crack statistics with a strain rate of 202.28 s^−1^. As shown in the figure, between 0 and ~12°, the total crack density was 883, and the total number of cracks in the interval of 12~24° was 845. It is worth noting that in the eight intervals of 0~96°, the total number of cracks showed a linear decrease trend, and in the interval of 96~180°, the number of cracks continued to increase, showing a linear growth trend. When the angle reached 180°, the number of cracks increased to a maximum value of 908. In general, under a single strain rate, the crushing mode of the specimen was the same as the above-mentioned failure mode.

**Figure 18:**
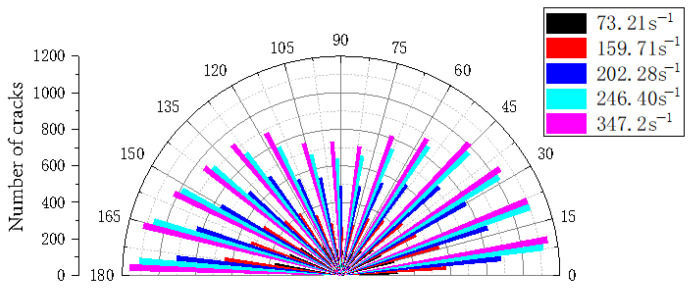
Crack distribution at different strain rates.

Figure 18 shows the number and angle of cracks in the numerical model of the reef limestone concrete at different strain rates. The figure clearly shows that as the strain rate increased, the crack density in each direction climbed linearly. When the strain rate increased from 73.21 s^−1^ to 246.40 s^−1^, the crack growth amplitude was the largest in this process. When the strain rate rose from 246.70 s^−1^ to 347.2 s^−1^, the crack growth amplitude was slow. This shows that when the strain rate exceeded 246.70 s^−1^, the model test coupon had almost been completely destroyed, and the model’s crushing diagram no longer changed dramatically.

## 4. Conclusions

Based on the continuous (FDM finite difference method)-discrete (DEM discrete element method) coupling method, a biaxial SHPB simulation system of reef limestone concrete was established. A half-sine wave was applied to the incident rod to perform a dynamic impact simulation. The correctness was verified by combining indoor experiments. Subsequent microscale analysis elucidated the dynamic behavior of reef limestone concrete. Key findings include the following:

(1) As the strain rate escalated, the dynamic stress peak initially exhibited rapid growth followed by stabilization, with a 25.51% enhancement observed. When the strain rate increased to 67.8 s^−1^, failure transitioned from mode I to mode II, accompanied by the disappearance of stress rebound phenomena. Dynamic stress–strain curves demonstrated progressive opening characteristics that intensified with the strain rate elevation.
(2) By programming the crack time-history monitoring program using the built-in FISH language, it was found that as the stress increased, the internal crack growth rate of the specimen was faster. When the stress decreased, the internal crack growth tended to be stable.
(3) The dynamic performance of the numerical model of reef limestone concrete under biaxial static load conditions is closely related to the magnitude of its initial static load. Under a constant σ_1_ static load, an elevating σ_2_ static load under biaxial conditions enhanced the σ_1_-direction dynamic peak stress while suppressing the σ_2_-direction dynamic peak stress. The stress constraint value in the σ_2_ direction had an inhibitory effect on the degree of sample crushing. With the increase in the stress constraint value, the number of model cracks decreased by 10% and 30%, respectively. The degree of internal crushing of the sample was significantly reduced. In the (σ_1_,10) stress constraint state, the constraint stress in the impact direction promoted the crushing of the sample, and the angle and number distribution of the cracks were M-shaped. This discovery can be directly applied to the optimization of key structures such as tunnel lining and breakwaters on islands and reefs; in areas with frequent dynamic loads, designing multi-directional constraint systems can significantly improve the impact toughness of concrete and extend the service life of the structure.

## Figures and Tables

**Table 1:** Physical properties of the reef tuff aggregates.

Coarse aggregate	Bulk density (kg/m^3^)	Apparent density (kg/m^3^)	Porosity %	Moisture content %	Water absorption %
	873	1939.6	59	13.2	22.9
Fine Aggregate	Bulk density (kg/m^3^)	Apparent density (kg/m^3^)	Fineness modulus	Moisture content %	Water absorption %
	1392	2698.5	2.5	2.9	3.7

**Table 2:** Reef tuff concrete proportions (kg/m^3^).

Name	Ordinary Portland Cement	Reef Limestone	Reef Limestone	Groundwater	Fly Ash	Slag	Anti-Cracking Waterproofing Agent
Dosage	780	700	300	250	70	150	15

**Table 3:** Indoor test results.

Impact Load/MPa	Strain Rate/s^−1^	Dynamic Peak Intensity/MPa	Peak Strain/%	Destruction Form
0.2	54.65	68.20	0.489	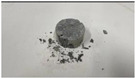
0.4	83.88	84.48	0.524	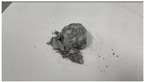
0.6	106.57	88	0.576	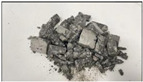
0.8	124.60	97.90	0.644	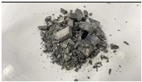

**Table 4:** Comparison between indoor test results and numerical simulation results.

Strain Rate		Sample Crushing State	Simulate Broken State	Dynamic Yield Strength	
Indoor Test	Numerical Simulation			Experiment (MPa)	Simulation (MPa)
83.88	81.2	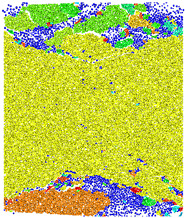	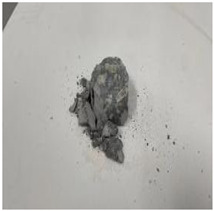	84.48	85
106.57	104.8	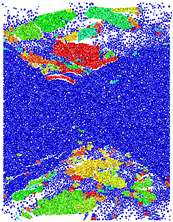	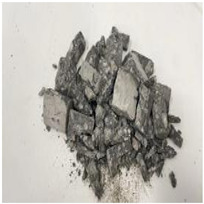	88	86.98

**Table 5:** Calibration table for the reef tuff concrete parameters.

Type	Contact Model	Particle Size	Coefficient of Friction	Density (kg/m^3^)	Elastic Modulus (GPa)	Stiffness Ratio (kratio)	Fj_ten (MPa)	Fj_coh (MPa)	Fj_fa (°)
Cement particles	Flat-joint model	0.45 × 10^−3^~0.74 × 10^−3^	0.2	2800	12.2	3.0	28.2	52.5	35
Aggregate particles	Flat-joint model		0.25	2500	15.0	3.0	40.0	70.0	45
ITZ	Flat-joint model		0.3	2800	10	3.0	30	40	35

**Table 6:** Calibration table for the rod system parameters.

Type	Contact Model	Density (kg/m^3^)	Rod Length (m)	Elastic Modulus (GPa)	Poisson’s Ratio
Incident rod	Elastic model	7800	2.0	205	0.25
Transmission rod	Elastic model	7800	1.5	205	0.3

## Data Availability

The original contributions presented in the study are included in the article, further inquiries can be directed to the corresponding author.
